# HIV Epidemiology, Care, and Treatment Outcomes Among Student and Nonstudent Youths Living With HIV in Southwest China Between 1996 and 2019: Historical Cohort Study

**DOI:** 10.2196/38881

**Published:** 2023-02-24

**Authors:** Na Wang, Guanghua Lan, Qiuying Zhu, Huanhuan Chen, Jinghua Huang, Qin Meng, Zhiyong Shen, Shujia Liang, Xiuling Wu, Liuhong Luo, Rongyi Ye, Jinli Chen, Shengkui Tan, Hui Xing, Yiming Shao, Yuhua Ruan, Mei Lin

**Affiliations:** 1 Guangxi Key Laboratory of Major Infectious Disease Prevention Control and Biosafety Emergency Response Guangxi Center for Disease Control and Prevention Nanning China; 2 Guangxi Key Laboratory of Environmental Exposomics and Entire Lifecycle Heath, School of Public Health Guilin Medical University Guilin China; 3 State Key Laboratory of Infectious Disease Prevention and Control National Center for AIDS/STD Control and Prevention, Chinese Center for Disease Control and Prevention Collaborative Innovation Center for Diagnosis and Treatment of Infectious Diseases Beijing China

**Keywords:** HIV, student, nonstudent, antiretroviral therapy, mortality, China

## Abstract

**Background:**

Nearly one-third of new HIV infections occurred among youth in 2019 worldwide. Previous studies suggested that student youths living with HIV and nonstudent youths living with HIV might differ in some risk factors, transmission routes, HIV care, and disease outcomes.

**Objective:**

This study aimed to compare the HIV epidemic, disease outcomes, and access to care among student and nonstudent youths living with HIV aged 16 to 25 years in Guangxi, China.

**Methods:**

We performed a historical cohort study by extracting data on all HIV or AIDS cases aged 16 to 25 years in Guangxi, China, during 1996-2019 from the Chinese Comprehensive Response Information Management System of HIV or AIDS. We conducted analyses to assess possible differences in demographic and behavioral characteristics, HIV care, and disease outcomes between student and nonstudent youths living with HIV. Multivariate Cox regression was used to assess differences in mortality and virologic failure between student and nonstudent cases.

**Results:**

A total of 13,839 youths aged 16 to 25 years were infected with HIV during 1996-2019. Among them, 10,202 cases were infected through sexual contact, most of whom were men (n=5507, 54%); 868 (8.5%) were students, and 9334 (91.5%) were not students. The number of student youths living with HIV was lower before 2006 but gradually increased from 2007 to 2019. In contrast, the nonstudent cases increased rapidly in 2005, then gradually declined after 2012. Student cases were mainly infected through homosexual contact (n=614, 70.7% vs n=1447, 15.5%; *P*<.001), while nonstudent cases were more likely to be infected through heterosexual contact (n=7887, 84.5% vs n=254, 29.3%; *P*<.001). Moreover, nonstudent cases had a significantly lower CD4 count than student cases at the time of HIV diagnosis (332 vs 362 cells/μL; *P*<.001). Nonstudents also had a delayed antiretroviral therapy (ART) initiation compared to students (93 days vs 22 days; *P*<.001). Furthermore, the mortality rate of 0.4 and 1.0 deaths per 100 person-years were recorded for student and nonstudent youths with HIV, respectively. Overall, the mortality risk in nonstudent cases was 2.3 times that of student cases (adjusted hazard ratio [AHR] 2.3, 95% CI 1.2-4.2; *P*=.008). The virologic failure rate was 2.3 and 2.6 per 100 person-years among student and nonstudent youths living with HIV, respectively. Nonstudent cases had double the risk of virologic failure compared to student cases (AHR 1.9, 95% CI 1.3-2.6; *P*<.001).

**Conclusions:**

Nonstudent youths living with HIV might face a low CD4 count at the time of HIV diagnosis, delayed ART initiation, and increased risk of death and virologic failure. Thus, HIV prevention and interventions should target youths who dropped out of school early to encourage safe sex and HIV screening, remove barriers to HIV care, and promote early ART initiation to curb the HIV epidemic among youths.

## Introduction

According to the Joint United Nations Programme on HIV/AIDS, 31% of new HIV infections globally occurred among youths aged 15 to 24 years in 2019 [1]. Sexual transmission, including heterosexual and homosexual transmission, was the primary route of HIV/AIDS transmission among young people [1-4]. In China, the incidence rate of HIV/AIDS increased fivefold from 0.27 cases per 100,000 in 2008 to 1.49 cases per 100,000 in 2017. HIV/AIDS is a major cause of death from infectious diseases in adolescents [5]. The number of youths living with HIV showed this increase in some cities [6]. Furthermore, nonstudents aged 15 to 24 years accounted for 81% of HIV cases compared to 19% of students in 2017 [7]. Students and nonstudents might differ in HIV risk and risk factors [8].

Antiretroviral therapy (ART), introduced globally since 1996, improves immune function by suppressing viral replication and dramatically declines the morbidity and mortality of people living with HIV [9]. ART early initiation—initiating ART as early as the day of HIV diagnosis—has numerous benefits, including reducing the HIV epidemic, improving the health status and quality of life, as well as extending the life expectancy of people living with HIV [10,11]. However, adolescents living with HIV in southern Africa are likely to have less access to HIV care and a hard time adhering to ART compared to adults, which in turn has resulted in negative consequences, such as poorer viral suppression and HIV-related deaths [12-14]. Student youths living with HIV may face more barriers to ART, such as stigma, fear of unintended disclosure of HIV status (due to the information on drug packaging and lack of privacy while taking pills), challenges in drug storage in school, inability to coordinate their studies and clinic-related activities, and lack of structured supporting systems in schools. These barriers may lead to sadness, anger, frustration, and stress among student youths living with HIV [15]. At the same time, the educational level, some demographic and behavioral characteristics, and low perceived risk for HIV are associated with late ART initiation in nonstudent youths living with HIV [6]. Thus, student and nonstudent youths with HIV aged between 16 and 25 years might also show differences in the ART initiation and treatment outcomes.

Currently, there is limited information on the demographic and behavioral characteristics, HIV infection routes, access to HIV care, long-term ART outcome, and mortality rate among student and nonstudent cases in a large population. Therefore, we conducted a historical cohort study from 1990 to 2019 to investigate the HIV epidemic, access to care, and HIV/AIDS disease outcomes among youths aged 16 to 25 years in Guangxi, China, to better understand the disease characteristics among student and nonstudent youths with HIV. The findings of this study will inform the design of AIDS programs, considering the differences in risk factors, transmission routes, the ART initiation rate, and treatment outcomes between students and nonstudents. In addition, the findings will guide more tailored HIV prevention and care strategies to curb the HIV epidemic among adolescents further.

## Methods

### Study Sites and Population

The study was conducted in Guangxi Zhuang Autonomous Region, southwest China. Guangxi was among the top 3 provinces and autonomous regions with the most HIV/AIDS cases in China from 2004 to 2007. Besides, it had the second highest number of HIV/AIDS cases between 2011 and 2013 [16]. Most HIV cases were infected through sexual contact [17], and youths were very vulnerable to HIV. Therefore, we designed this study to investigate HIV-positive youths aged 16 to 25 years (age at diagnosis) and living in Guangxi (at the time of data exporting, January 2021) between January 1, 1996, and December 31, 2020.

### Data Sources

We used data extracted from the Chinese Comprehensive Response Information Management System of HIV/AIDS (CRIMS), which includes the National Case Reporting Database (NCRD) and the National Free Antiretroviral Therapy Database (NFATD). The NCRD contains data on all HIV/AIDS cases from local hospitals, Centers for Disease Control and Prevention (CDC) clinics, and blood banks, including demographic information, the HIV diagnosis date, reporting organization, as well as the first CD4 test reporting date and result. The first CD4 test was conducted after an HIV diagnosis. The NFATD contains information on the ART, such as viral load and time, ART regimens, interregional transfer, comorbid diseases, loss to follow-up, death, and time of death [18]. In our study, time for the initiation of the ART was used as the baseline time point, and then patients were invited to visit the clinic for follow-up at 15 days, 1 and 3 months, and every 3 months; their information about the ART was then updated in the NFATD accordingly. Patients were also recommended to test the viral load and CD4 count once every 12 months. Individual cases in the NFATD were matched to the NCRD database using the case’s card numbers [18]. Anonymous and deidentification data were extracted from the NCRD and NFATD by designated staff at CDC offices in Guangxi.

Variables collected in this study are about the patient status at the time of HIV diagnosis and follow-up information, including whether they are a student or nonstudent, age, gender, ethnicity, marital status, education, time and sites of HIV diagnosis, infection route, CD4 count, the ART initiation, HIV viral load, and death. We collected the HIV-positive cases reported from January 1, 1996, to December 31, 2019. However, the viral load and death data were collected until December 31, 2020. This study included all newly diagnosed HIV-positive youths aged 16 to 25 years (age at diagnosis) each year living in Guangxi.

### Ethics Approval

This study was approved by the institutional review board of Guangxi (GXIRB2016-0047-3).

### Data Analyses

We compared the demographic and behavioral characteristics, HIV care, virological loads, and deaths between students and nonstudents living with HIV. Chi-square tests were used for categorical variables (eg, gender, ethnicity, occupation, marital status, education, infection route, calendar year of HIV diagnosis, health facilities providing HIV diagnosis, and the ART initiation), and *t* tests or nonparametric tests were used for continuous variables (eg, CD4 count at HIV diagnosis and time from HIV diagnosis until the ART initiation). Multivariable Cox regression was conducted to assess if students or nonstudents’ status were associated with all-cause death and virologic failure, adjusting for sex, ethnicity, marital status, education, infection route, CD4 count before the ART initiation, and the World Health Organization (WHO) clinical stage before the ART.

## Results

### Demographic and Behavioral Characteristics Among Youths Living With HIV

**Figure 1:**
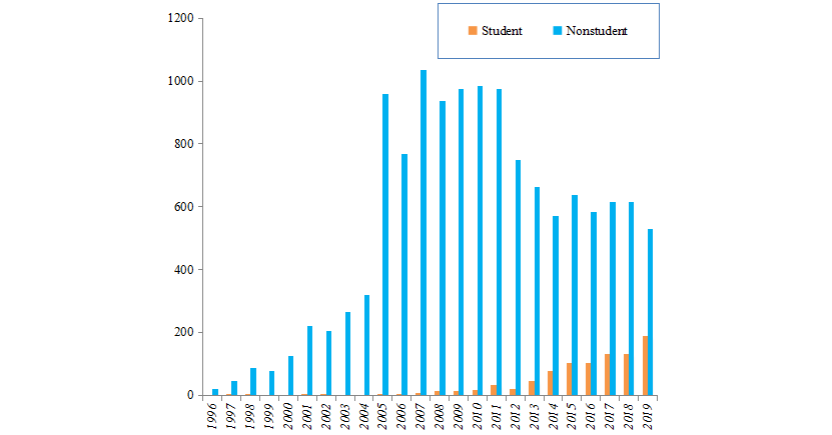
Annual cases of student and nonstudent youths living with HIV in Guangxi, China, from 1996 to 2019.

**Figure 2:**
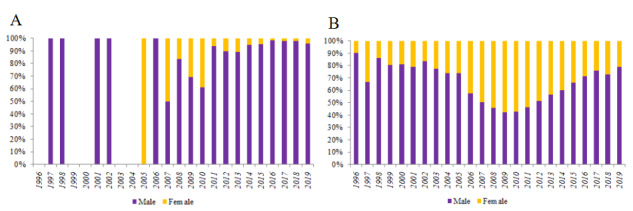
The gender of student (A) and nonstudent (B) youths living with HIV in Guangxi, China from 1996 to 2019.

**Figure 3:**
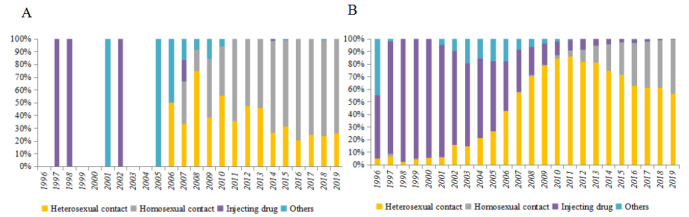
The infection routs of student(A) and nonstudent(B) youths living with HIV in Guangxi, China during 1996-2019.

**Figure 4:**
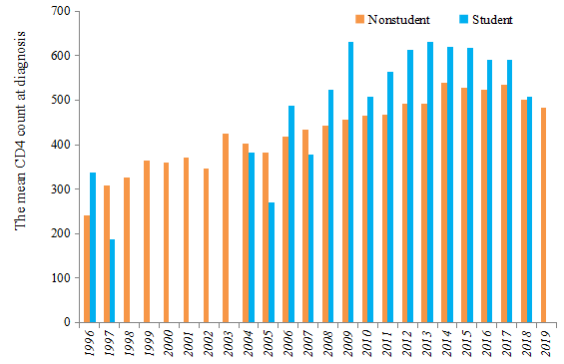
The mean CD4 count at diagnosis of student and nonstudent youths living with HIV in Guangxi, China from 1996 to 2019.

A total of 13,839 youths aged 16 to 25 years were diagnosed with HIV or AIDS in Guangxi between 1996 and 2019. Among them, 883 (6.4%) were students, and 12,956 (93.6%) were nonstudents. The number of students living with HIV was low before 2006 but gradually increased from 2007 to 2019. In contrast, the nonstudent cases living with HV increased rapidly in 2005 and gradually declined after 2012. The number of student youths was one-third of the nonstudents youths living with HIV in 2019 (Figure 1). The student cases were predominantly male; the majority of nonstudent youths living with HIV were male except during 2008-2011 (Figure 2). The homosexual transmission was the main infection route among student youths living with HIV in Guangxi. However, among the nonstudent cases, injecting drugs was the main route of infection before 2005, followed by heterosexual transmission during 2006-2019. At the same time, homosexual transmission showed an increasing trend in recent years (Figure 3). The mean CD4 count at the time of diagnosis was slightly higher in student youths living with HIV and showed an increasing trend during 1996-2019 in both student and nonstudent youths living with HIV (Figure 4).

Of the 13,839 youths, 61.8% (n=8551) were male and 38.2% (n=5288) were female. The HIV-positive youths were mostly of Han ethnicity (n=8647, 62.5%), nonfarmer (n=8687, 62.8%), single (n=8768, 63.4%), and with middle school education level or below (n=9211, 66.6%). Homosexual contact was the primary route of transmission for students (n=614, 69.5%), while heterosexual contact was the main transmission route for nonstudents (n=7887, 60.9%; *P*<.001; Table 1).

**Table 1:** Demographic and behavioral characteristics of youths living with HIV aged 16-25 years by student status in Guangxi, China, from 1996 to 2019.

Characteristics		Total, n (%)	Students, n (%)	Nonstudents, n (%)	*P* value	
Total		13,839 (100)	883 (6.4)	12,956 (93.6)		
**Gender**						<.001
	Male	8551 (61.8)	832 (94.2)	7719 (59.6)		
	Female	5288 (38.2)	51 (5.8)	5237 (40.4)		
**Ethnicity**						<.001
	Han	8647 (62.5)	601 (68.1)	8046 (62.1)		
	Other	5192 (37.5)	282 (31.9)	4910 (37.9)		
**Occupation**						<.001
	Farmer	5152 (37.2)	0 (0)	5152 (39.8)		
	Other	8687 (62.8)	883 (100)	7804 (60.2)		
**Marital status**						<.001
	Single	8768 (63.4)	875 (99.1)	7893 (60.9)		
	Currently married	3962 (28.6)	5 (0.6)	3957 (30.5)		
	Divorced, separated, or widowed	526 (3.8)	0 (0)	526 (4.1)		
	Unknown	583 (4.2)	3 (0.3)	580 (4.5)		
**Education**						<.001
	Middle school and below	9211 (66.6)	22 (2.5)	9189 (70.9)		
	High school	1954 (14.1)	195 (22.1)	1759 (13.6)		
	College and above	1739 (12.6)	661 (74.9)	1078 (8.3)		
	Unknown	935 (6.8)	5 (0.6)	930 (7.2)		
**Infection route**						<.001
	Heterosexual contact	8141 (58.8)	254 (28.8)	7887 (60.9)		
	Homosexual contact	2061 (14.9)	614 (69.5)	1447 (11.2)		
	Injecting drug	2960 (21.4)	5 (0.6)	2955 (22.8)		
	Other	677 (4.9)	10 (1.1)	667 (5.1)		
**Calendar year of HIV diagnosis**						<.001
	1996-2003 (pre-ART^a^)	958 (6.9)	4 (0.5)	954 (7.4)		
	2004-2007 (no standardized ART)	3050 (22)	9 (1)	3041 (23.5)		
	2008-2012 (standardized ART)	4787 (34.6)	93 (10.5)	4694 (36.2)		
	2013-2019 (TDF^b^ added)	5044 (36.4)	777 (88)	4267 (32.9)		

^a^ART: antiretroviral therapy.

^b^TDF: tenofovir disoproxil fumarate.

### Demographic and Behavioral Characteristics Among Youths Living With HIV Through Sexual Contact

A total of 10,202 youths aged 16-25 years were infected with HIV through sexual contact between 1996 and 2019 in Guangxi. Among them, 8.5% (868/10,202) were students, and 91.5% (9334/10,202) were nonstudents. Compared with nonstudent cases, student cases were more likely to be male (n=820, 94.5% vs n=4687, 50.2%; *P*<.001), nonfarmer (n=868, 100% vs n=5396, 57.8%; *P*<.001), single (n=862, 99.3% vs n=5555, 59.5%; *P*<.001), with an educational level of college or above (n=656, 75.6% vs n=1066, 11.4%; *P*<.001). Furthermore, student youths living with HIV were more likely to be infected through homosexual contact (n=614, 70.7% vs n=1447, 15.5%; *P*<.001) and diagnosed with HIV between 2013 and 2019 (n=774, 89.2% vs n=4134, 44.3%; *P*<.001; Table 2).

**Table 2:** Demographic and behavioral characteristics of youths aged 16-25 years living with HIV through sexual contact by student status in Guangxi, China, from 1996 to 2019.

Characteristics			Total, n (%)		Students, n (%)		Nonstudents, n (%)		*P* value	
Total			10,202 (100)		868 (8.5)		9334 (91.5)			
**Gender**										<.001
	Male	5507 (54)		820 (94.5)		4687 (50.2)				
	Female	4695 (46)		48 (5.5)		4647 (49.8)				
**Ethnicity**										<.001
	Han	6319 (61.9)		594 (68.4)		5725 (61.3)				
	Other	3883 (38.1)		274 (31.6)		3609 (38.7)				
**Occupation**										<.001
	Farmer	3938 (38.6)		0 (0)		3938 (42.2)				
	Other	6264 (61.4)		868 (100.0)		5396 (57.8)				
**Marital status**										<.001
	Single	6417 (62.9)		862 (99.3)		5555 (59.5)				
	Currently married	3320 (32.5)		5 (0.6)		3315 (35.5)				
	Divorced, separated, or widowed	404 (4)		0 (0)		404 (4.3)				
	Unknown	61 (0.6)		1 (0.1)		60 (0.6)				
**Education**										<.001
	Middle school and below	6577 (64.5)		19 (2.2)		6558 (70.3)				
	High school	1834 (18)		193 (22.2)		1641 (17.6)				
	College and above	1722 (16.9)		656 (75.6)		1066 (11.4)				
	Unknown	69 (0.7)		0 (0)		69 (0.7)				
**Infection route**										<.001
	Heterosexual contact	8141 (79.8)		254 (29.3)		7887 (84.5)				
	Homosexual contact	2061 (20.2)		614 (70.7)		1447 (15.5)				
**Calendar year of HIV diagnosis**										<.001
	1996-2003 (pre-ART^a^)	92 (0.9)		0 (0)		92 (1)				
	2004-2007 (no standardized ART)	1227 (12)		5 (0.6)		1222 (13.1)				
	2008-2012 (standardized ART)	3975 (39)		89 (10.3)		3886 (41.6)				
	2013-2019 (TDF^b^ added)	4908 (48.1)		774 (89.2)		4134 (44.3)				

^a^ART: antiretroviral therapy.

^b^TDF: tenofovir disoproxil fumarate.

### HIV Care Between Student and Nonstudent Youths Living With HIV

Among the HIV/AIDS cases infected through sexual contact reported from 2008 to 2019 in Guangxi, student youths living with HIV were more likely to receive an HIV diagnosis in CDC compared to nonstudent cases (n=458, 59.6% vs n=3099, 44.1%; *P<*.001). In addition, student cases had a higher average CD4 count (median 362 [IQR 262-481] cells/μL) compared to nonstudents (median 332 [IQR 204-459] cells/μL; *P<*.001) at diagnosis. Although most youths were on the ART in both groups, student youths with HIV who were on the ART were significantly higher in number compared to nonstudent youths (n=740, 96.2% vs n=6247, 88.8%; *P<*.001). The median time from HIV diagnosis until the ART initiation was 22 days for student cases and 93 days for nonstudent cases (*P<*.001; Table 3).

**Table 3:** HIV care of youths living with HIV who were infected through sexual contact (aged 16-25 years) by student status in Guangxi, China, from 2008 to 2019.

HIV care		Student (n=769)	Nonstudent (n=7034)	*P* value
**Health facilities providing HIV diagnosis, n (%)**				<.001
	Hospital	270 (35.1)	3710 (52.7)	
	CDC^a^	458 (59.6)	3099 (44.1)	
	Other	41 (5.3)	225 (3.2)	
CD4 count at HIV diagnosis (cells/μL), median (IQR)		362 (262-481)	332 (204-459)	<.001
**On ART, n (%)**				<.001
	Yes	740 (96.2)	6247 (88.8)	
	No	29 (3.8)	787 (11.2)	
**Time from HIV diagnosis until the ART^b^ initiation by CD4 count (day), median (IQR)**				
	Overall (n=7803)	22 (10-85)	93 (16-979)	<.001
	0-199 (n=2768)	26 (11-164)	151 (21-1349)	<.001
	200-349 (n=2597)	20 (9-88)	80 (15-789)	<.001
	≥350 (n=2291)	24 (11-74)	67 (14-739)	<.001
	Never tested (n=147)	24 (18-57)	267 (18-1065)	.04

^a^CDC: Centers for Disease Control and Prevention.

^b^ART: antiretroviral therapy.

### Mortality Rate Between Student and Nonstudent Youths Living With HIV

Among the 7803 youths infected through sexual contact and who started the ART from 2008 to 2019, a total of 45,610.4 person-years were observed, and 446 youths died before December 31, 2020. The overall death rate among nonstudent youths living with HIV was 1.0 deaths per 100 person-years, which was higher than that among student youths living with HIV (0.4 deaths per 100 person-years). After adjusting for sex, ethnicity, marital status, education, infection route, CD4 count before the ART initiation, and the WHO clinical stage before the ART, nonstudent youths living with HIV had mortality risk doubled compared to student youths with HIV (adjusted hazard ratio [AHR] 2.3, 95% CI 1.2-4.2; *P*=.008). Youths who had initiated a zidovudine-based regimen had a 30% decreased mortality rate compared to those who took a stavudine-based regimen (AHR 0.7, 95% CI 0.5-0.9; *P*=.002). Youths who initiated a tenofovir disoproxil fumarate–based regimen also had a decreased mortality rate compared to the stavudine-based regimen group (AHR 0.7, 95% CI 0.5-1.0; *P*=.03; Table 4).

**Table 4:** Mortality rate of youths living with HIV aged 16-25 years by student status in Guangxi, China, from 2008 to 2019.

Characteristics		Patients, n	Deaths, n	Person-years, n	Deaths per 100 person-years (95% CI)	HR^a^ (95% CI)	*P* value	AHR^b^ (95% CI)	*P* value
Overall		7803	446	45,610.4	1.0 (0.9-1.1)				
**Age group (16-25 years)**							<.001		.008
	Student	769	11	2976.3	0.4 (0.2-0.7)	1.0		1.0	
	Nonstudent	7034	435	42,634.2	1.0 (0.9-1.1)	3.1 (1.7-5.7)		2.3 (1.2-4.2)	
**Calendar year of initiating the ART^c^**							.003		.44
	2008-2012	2417	229	22,653.7	1.0 (0.9-1.1)	1.0		1.0	
	2013-2019	5386	217	22,956.8	0.9 (0.8-1.1)	0.7 (0.6-0.9)		1.1 (0.9-1.5)	
**Initial regimen**									
	Stavudine-based	637	111	5606.3	2.0 (1.6-2.4)	1.0	—^d^	1.0	—
	Zidovudine-based	3274	174	23,438.3	0.7 (0.6-0.9)	0.4 (0.3-0.5)	<.001	0.7 (0.5-0.9)	.002
	TDF^e^-based	3724	154	15,170.1	1.0 (0.9-1.2)	0.4 (0.3-0.5)	<.001	0.7 (0.5-1.0)	.03
	Other	168	7	1395.7	0.5 (0.2-1.1)	0.3 (0.1-0.5)	<.001	0.3 (0.2-0.7)	.005

^a^HR: hazard ratio.

^b^AHR: adjusted hazard ratio; adjusted for sex, ethnicity, marital status, education, infection route, CD4 count before the antiretroviral therapy (ART) initiation, and the World Health Organization (WHO) clinical stage before the ART.

^c^ART: antiretroviral therapy.

^d^Not applicable.

^e^TDF: tenofovir disoproxil fumarate.

### Virologic Failure Between Student and Nonstudent Youths Living With HIV

We assessed the virologic failure among youths who started ART in 2008 since free and standardized ART programs started from 2008 in China. Of the 4119 youths who started ART during 2008-2019, a total of 24,388.6 person-years were observed, and 632 youths met the clinical criteria of virologic failure during the follow-up period. The virologic failure rate among nonstudent youths with HIV was 2.6 deaths per 100 person-years, which was higher than that of student youths with HIV (2.3 per 100 person-years). After being adjusted for sex, ethnicity, marital status, education, infection route, CD4 count before the ART initiation, and the WHO clinical stage before the ART, the risk of virologic failure among nonstudent youths with HIV had doubled compared to student youths (AHR 1.9, 95% CI 1.3-2.6; *P*<.001). Youths who had initiated the ART during 2013-2019 had a 30.9 times increased risk of virologic failure compared with those who initiated the ART during 2008-2012 (AHR 31.9, 95% CI 18.2-55.9; *P*<.001). Youths who initiated the ART with a zidovudine-based (AHR 1.5, 95% CI 1.2-2.0; *P*=.003) and TDF-based regimen (AHR 1.9, 95% CI 1.4-2.6; *P*<.001) had an increased risk of virologic failure compared to those who took a stavudine-based regimen (Table 5).

**Table 5:** Virologic failure (≥400 copies/mL) of youths living with HIV aged 16-25 years by student status in Guangxi, China, from 2008 to 2019.

Characteristics		Patients, n	Virologic failure, n	Person-years, n	Virologic failure per 100 person-years (95% CI)	HR^a^ (95% CI)	*P* value	AHR^b^ (95% CI)	*P* value
Overall		4199	632	24,388.6	2.6 (2.4-2.8)				
**Age group (16-25 years)**							.01		<.001
	Student	428	38	1664.2	2.3 (1.7-3.1)	1.0		1.0	
	Nonstudent	3691	594	22,724.4	2.6 (2.4-2.8)	0.7 (0.5-0.9)		1.9 (1.3-2.6)	
**Calendar year of initiating the ART^c^**							<.001		<.001
	2008-2012	1247	270	12,004.0	2.3 (2.0-2.5)	1.0		1.0	
	2013-2019	2872	362	12,384.6	2.9 (2.6-3.2)	46.7 (28.1-77.4)		31.9 (18.2-55.9)	
**Initial regimen**									
	Stavudine-based	325	75	3034.0	2.5 (2.0-3.1)	1.0	—^d^	1.0	—
	Zidovudine-based	1658	304	12,168.0	2.5 (2.2-2.8)	1.6 (1.2-2.0)	<.001	1.5 (1.2-2.0)	.003
	TDF^e^-based	2052	241	8543.4	2.8 (2.5-3.2)	5.0 (3.7-6.7)	<.001	1.9 (1.4-2.6)	<.001
	Other	84	12	643.1	1.9 (1.1-3.3)	0.6 (0.3-1.1)	.08	0.5 (0.3-1.0)	.05

^a^HR: hazard ratio.

^b^AHR: adjusted hazard ratio; adjusted for sex, ethnicity, marital status, education, infection route, CD4 count before the ART initiation, and the WHO clinical stage before the ART.

^c^ART: antiretroviral therapy.

^d^Not applicable.

^e^TDF: tenofovir disoproxil fumarate.

## Discussion

To our knowledge, this is the first study investigating the characteristics of youths living with HIV in southwest China. Nonstudent youths living with HIV accounted for 93.6% (12956/13839) of the total HIV cases in youths during 1996-2019. This percentage was much higher than that in youths aged 15-24 years in Hangzhou, China, during 2012-2016 (70.4%) [6]. Given the low numbers before 2006, an obvious increasing trend was observed among student youths living with HIV, approximately one-third of nonstudent cases in 2019. Homosexual transmission was the main infection route among student youths living with HIV in Guangxi (n=614, 69.5%), while heterosexual contact was the primary transmission route among nonstudent cases (n=7887, 60.9%), which was different from the primary transmission route in Hangzhou (ie, homosexual transmission) [6]. Heterosexual contact is the primary mode of HIV transmission in China [7,19]. Although the mean CD4 count at the time of diagnosis was slightly higher in student cases and consistently increased from 1996 to 2019 among student and nonstudent youths living with HIV, there is still a need to promote frequent HIV testing for early HIV diagnosis in youths in the future.

Among youths who were infected with HIV through sexual contact, the 2 groups showed differences: student youths with HIV were mainly male (n=820, 94.5% vs n=4687, 50.2%) and unmarried (n=862, 99.3%), and homosexual contact was the primary transmission route (n=614, 70.7%). In general, youths were considered sexually active and less likely to use a condom [20]. Unprotected sexual activity raises the risk of HIV transmission among youths [21]. Moreover, our study demonstrated that most nonstudent youths living with HIV had an educational level of middle school or below (n=6558, 70.3%). Limited access to sexual education, leading to the lack of HIV awareness, could be a possible reason of high-risk sexual behaviors. Thus, these findings could be applied in tailored HIV prevention programs with the goal of reducing the HIV epidemic among youths. In addition, more attention should be given to homosexual student and nonstudent youths who dropped out before high school.

Furthermore, student youths living with HIV had declined CD4 levels at the time of HIV diagnosis, and the CD4 level for nonstudent cases was even lower. This might be a result of delayed HIV testing. Besides, more than half of the nonstudents with HIV (n=3710, 52.7%) received an HIV diagnosis in hospitals instead of CDCs, implying low awareness of HIV testing, as CDCs have voluntary counseling and testing, but clinics and hospitals usually conduct HIV tests as part of physical examination. Moreover, student youths with HIV (n=740, 96.2%) had a slightly higher rate of the ART compared to nonstudent cases. At the same time, nonstudents had a longer median time for the ART initiation (93 days) compared to students (22 days). Stigma and lack of knowledge are major factors leading to delayed ART [6]. Therefore, future programs should promote early diagnosis and treatment to enhance the treatment efficacy and improve outcomes [11], and consequently, reduce morbidity and mortality [22].

Nonstudnet youths living with HIV had a higher risk of experiencing virologic failure and death compared with student cases after the ART initiation. More attention should be given to nonstudents for preventing virologic failure and death. It could be explained by the findings of this study that nonstudent youths with HIV in Guangxi were less likely to adopt the ART, had a lower CD4 count at the time of HIV diagnosis, and a delayed ART initiation. Early ART initiation could lead to improved clinical outcomes [23]. Therefore, future programs should work on eliminating barriers to HIV care and promoting early ART initiation among youths, especially among nonstudents. In this study, 70.9% (n=9189) of nonstudents youths with HIV in Guangxi had an educational level of middle school or below, and only 8.3% (n=1078) obtained university or higher education. Previous studies found that low level of education is associated with delayed HIV care [24], and school-based interventions could increase HIV knowledge and awareness among students [25,26], which in turn could probably reduce their risk behaviors and promote benefit behaviors. In addition to the school-based interventions, various interventions targeting out-of-school youths should be promoted to increase ART awareness and reduce risky sexual behaviors among the youths.

Based on the findings of this study, tailored HIV programs could be more effective in HIV prevention among the youths. For nonstudents aged 16-25 years, who accounted for the majority of HIV cases, social media–oriented HIV education using platforms like Douyin and WeChat has a great potential to deliver future HIV programs due to their wide coverage and accessibility. For students, more attention could be given to the high-risk groups, such as homosexual men. At the same time, sex education and safe sex should be strengthened in middle school, high school, and college. Given the high mortality rate as well as high risk of virologic failure and delayed ART among nonstudent youths with HIV, increased health protection awareness is crucial to encourage early diagnosis and early ART initiation [27]. Moreover, peer counseling and group therapy might be good ways to address psychological barriers to HIV testing and care, including stigma and lack of support [28,29]. Furthermore, HIV prevention campaigns could also create more awareness among the general public and clinic doctors, reduce stigma, and create a safe environment for youths to discuss their behaviors [30].

However, this study had some limitations. First, the study used historical cohort study data extracted from existing public health databases, and some information that might be useful were not available, such as the details on ART adherence and its side effects. Second, we investigated all-cause mortality instead of AIDS-related mortality, which might give different results. Third, students and nonstudents were generalized, disregarding their level of education; for example, middle and high school students might have different behaviors in HIV care and treatment outcomes from college students. For nonstudents, different levels of education may result in different perceptions and concerns about health. Thus, we adjusted for the educational background in the multivariate analysis to correct these discrepancies. Despite these limitations, this study provides valuable information on HIV risk behaviors, HIV care, and treatment outcomes for both student and nonstudent youths living with HIV. Overall, these findings will guide future HIV prevention and intervention programming in youths.

## Abbreviations

AHR: adjusted hazard ratio
ART: antiretroviral therapy
CDC: Centers for Disease Control and Prevention
CRIMS: Chinese Comprehensive Response Information Management System
NCRD: National Case Reporting Database
NFATD: National Free Antiretroviral Therapy Database
WHO: World Health Organization
